# Genome-scale meta-analysis of breast cancer datasets identifies promising targets for drug development

**DOI:** 10.1186/s40709-021-00136-7

**Published:** 2021-02-16

**Authors:** Reem Altaf, Humaira Nadeem, Mustafeez Mujtaba Babar, Umair Ilyas, Syed Aun Muhammad

**Affiliations:** 1 Department of Pharmaceutical Chemistry, Faculty of Pharmaceutical Sciences, Riphah International University, Islamabad, 44000 Pakistan; 2 Shifa College of Pharmaceutical Sciences, Shifa Tameer-E-Millat University, Islamabad, 44000 Pakistan; 3 Department of Pharmaceutics, Faculty of Pharmaceutical Sciences, Riphah International University, Islamabad, 44000 Pakistan; 4 Institute of Molecular Biology and Biotechnology, Bahauddin Zakariya University, Multan, 66000 Pakistan

**Keywords:** Breast cancer, Microarray datasets, Pathway enrichment analysis, Gene ontology, miRNA, Drug-gene network

## Abstract

**Background:**

Because of the highly heterogeneous nature of breast cancer, each subtype differs in response to several treatment regimens. This has limited the therapeutic options for metastatic breast cancer disease requiring exploration of diverse therapeutic models to target tumor specific biomarkers.

**Methods:**

Differentially expressed breast cancer genes identified through extensive data mapping were studied for their interaction with other target proteins involved in breast cancer progression. The molecular mechanisms by which these signature genes are involved in breast cancer metastasis were also studied through pathway analysis. The potential drug targets for these genes were also identified.

**Results:**

From 50 DEGs, 20 genes were identified based on fold change and *p*-value and the data curation of these genes helped in shortlisting 8 potential gene signatures that can be used as potential candidates for breast cancer. Their network and pathway analysis clarified the role of these genes in breast cancer and their interaction with other signaling pathways involved in the progression of disease metastasis. The miRNA targets identified through miRDB predictor provided potential miRNA targets for these genes that can be involved in breast cancer progression. Several FDA approved drug targets were identified for the signature genes easing the therapeutic options for breast cancer treatment.

**Conclusion:**

The study provides a more clarified role of signature genes, their interaction with other genes as well as signaling pathways. The miRNA prediction and the potential drugs identified will aid in assessing the role of these targets in breast cancer.

## Background

Cancer is one of the leading causes of death for the past several years and is the second cause of mortality according to the American Cancer Society (ACS) statistics after cardiovascular, infectious and parasitic disorders. Breast cancer is one of the most commonly diagnosed life-threatening malignancy that remains to be the leading cause of cancer incidence and mortality in women globally [1].

Several factors have been attributed towards the development of breast carcinoma. These include age, personal history of breast cancer, reproductive, environmental and genetic factors. Increasing age enhances the risk of breast cancer development [2]. Having a personal history of breast cancer also contributes towards a greater risk of second breast cancer that can be ipsilateral or contralateral. Family history of breast cancer can also enhance the risk of development of cancer in women. About 5–10% of women with breast cancer show an autosomal dominant inheritance while 20–25% have a positive family history [3]. Genetic predisposition alleles showing 40–85% of lifetime threat of breast cancer development include BRCA1 and BRCA2 mutations, TP53 mutations, PTEN, STK11, E-cadherin and neurofibromatosis (NF) [4].

The treatment strategies for breast cancer are largely determined by the status of progesterone receptor, estrogen receptor and the human epidermal growth factor receptor 2. Clinicopathological factors such as tumor grade, size and status of lymph node also determine the therapeutic plan, however, the biomarkers for the tumor invasion and metastasis are of profound importance in order to formulate new markers and treatment strategies for breast carcinomas. This will aid in both current therapies and tumor prognosis [5].

With the aid of in silico bioinformatic approaches the attainment of new treatment strategies have become easier. One such approach that has helped in identifying new markers in cancer therapy is the cDNA differential analysis [6]. In this study, 24 datasets were downloaded to analyze gene expression profiles in breast cancer and a functional analysis was performed to identify the differentially expressed genes (DEGs) between breast tumor cells and treated tissues. A genetic network was constructed as well as pathway analysis and miRNA target identification were performed to understand the underlying molecular mechanisms and to identify potential therapeutic targets for breast cancer. Moreover, drug-gene network analysis has also been performed to identify potential drug targets for breast cancer.

## Methods

### Accession of gene expression data

The study focuses on the identification of potential breast cancer targets through a differential screening method. The datasets of breast cancer were accessed from Gene Expression Omnibus database. The screening criteria was “organism: *Homo sapiens*”, and “experiment type: expression profiling by array”. The Affymetrix GeneChip Human Genome U133 Plus 2.0 Array (CDF: Hs133P_Hs_ENST, version 10) (Affymetrix, Inc., Santa Clara, CA, 95051, USA) platform was used. All datasets comprised of GEO accession number, platform, sample type, number of samples and gene expression data. The array platform and hgu133plus2 annotation platform of probes were used to identify the differentially expressed genes. The software R and Bioconductor packages AffyQCReport, Affy, Annotate, AnnotationDbi, Limma, Biobase, AffyRNADegradation, hgu133plus2cdf, and hgu133a2cdf were used to perform the computational analysis [7].

### Preprocessing and differential expression analysis of microarray datasets

The preprocessing of datasets was performed by preparing the phenodata files for each dataset in a recognizable format [8]. Using the R version 3.1.3, the Bioconductor ArrayQuality Metrics package was utilized for the normalization of the data to a median expression level for each gene [7]. After normalization, the background correction was done for perfect match (pm) and mismatch (mm) by Robust Multi-array Analysis (RMA). The method was used to eliminate the artifacts and local noise. The expression value with a *p*-value < 0.15 was measured as marginal log transformation. Afterwards, summarization was performed by RMA-algorithm in order to measure the averages between probes in a probe set to attain the summary of intensities.

The quality of RNA in these microarray datasets was measured using the AffyRNADegradation package of Bioconductor, also called degradation analysis [9]. Lastly, the DEGs in each dataset were identified by pairwise comparison and the Benjamini–Hochberg method [10] was employed for multiple testing correction. The differentially expressed genes were shortlisted and ranked according to their *p*-values and resulting scores. The cutoff values set were *p*-value ≤ 0.05, FDR < 0.05 (False Discovery Rate) and absolute log fold change logFC > 1 [11] to calculate the moderated statistics.

### Data curation and cluster analysis

The shortlisted genes obtained through differential expression analysis were further screened to confirm their role in breast cancer using diverse data sources such as PubMed (http://www.ncbi.nlm.nih.gov/pubmed), MeSH (http://www.ncbi.nlm.nih.gov/mesh), OMIM (Online Mendelian Inheritance in Man) (http://www.ncbi.nlm.nih.gov/omim), and PMC database (http://www.ncbi.nlm.nih.gov/pmc) [12]. Biomedical text mining helped in filtering significant disease specific genes. The CIMminner tool was used to perform the cluster analysis based on the expression values in each dataset using the Absolute Pearson correlation analysis. The cluster analysis revealed variations in gene expression levels between control and treated replicates [13].

### Network analysis and identification of gene signatures

The protein–protein interaction network helped in identifying the interaction of each protein with other genes having different biological or molecular functions in a diseased state as compared to normal. The Search Tool for the Retrieval of Interacting Genes/Proteins (STRING) [14] and Human Annotated and Predicted Protein Interaction (HAPPI) databases [15] were used to evaluate the proteins that interacted with each other in breast cancer with a confidence score of 0.999. The visualization and analysis of molecular interactions of seeder genes with the target genes were done using Cytoscape (version 3.2.1, Temple Place, Suite 330, Boston, MA 02111-1307 USA) software. The role of target genes in breast cancer was mapped by OMIM, MeSH, and PMC databases to identify the breast cancer associated gene signatures whose dysregulation causes a pathological phenotype. A molecular sub-network of those genes that were associated with pathways of interest causing breast cancer was constructed. The topological network properties were calculated using Network Analyzer in Cytoscape [16]. The web-based tools Database for Annotation Visualization and Integrated Discovery (DAVID) [17] and FunRich [18] were used to study the biological functions of these genes including the gene ontology, functional annotation and pathway enrichment analysis [19, 20].

### miRNA target prediction

miRNAs are small non-coding RNAs considered as post-transcriptional regulators of several biological processes. Dysregulation of miRNAs leads to disruption of signaling pathways causing disease. The influence of miRNAs on gene targets is one beneficial approach to get a better understanding of disease etiology [21]. The miRNA targets of breast cancer related genes were predicted by miRDB target predictor (www.mirdb.org), an online database for miRNA target prediction and functional annotation. The miRNAs were selected based on the target score (≤ 99).

### Integrated pathway modeling

The integrated and metabolic networks of breast cancer related source genes were analyzed and the correlation between test genes was observed. To recognize the underlying pathways involved in the progression of breast cancer, pathway analysis was performed for identifying biomarkers of the disease. The curation and mapping of candidate biomarkers were done using Kyoto Encyclopedia of Genes and Genomes (KEGG) [22], Reactome and Wiki pathways. PathVisio3tool was used to reconstruct the cellular and signaling pathways of potential biomarkers [23] and the potential mechanism of each marker in the pathway was studied based on evidence available in literature and databases.

### Drug-gene network analysis

The target genes interrelated with the anti-breast cancer drugs were identified using CTD (http://ctdbase.org/) database, an open source database for the curation of chemical–gene, gene–disease and chemical–disease interactions from literature [24]. The chemical–gene interaction query was used to access drugs against each breast cancer related genes. Drugs that were directly linked with breast cancer related genes were sorted in this interaction network. The FDA approval status of these drugs was also verified using the DrugBank database [25].

## Results

### Gene expression analysis and normalization

**Fig. 1:**
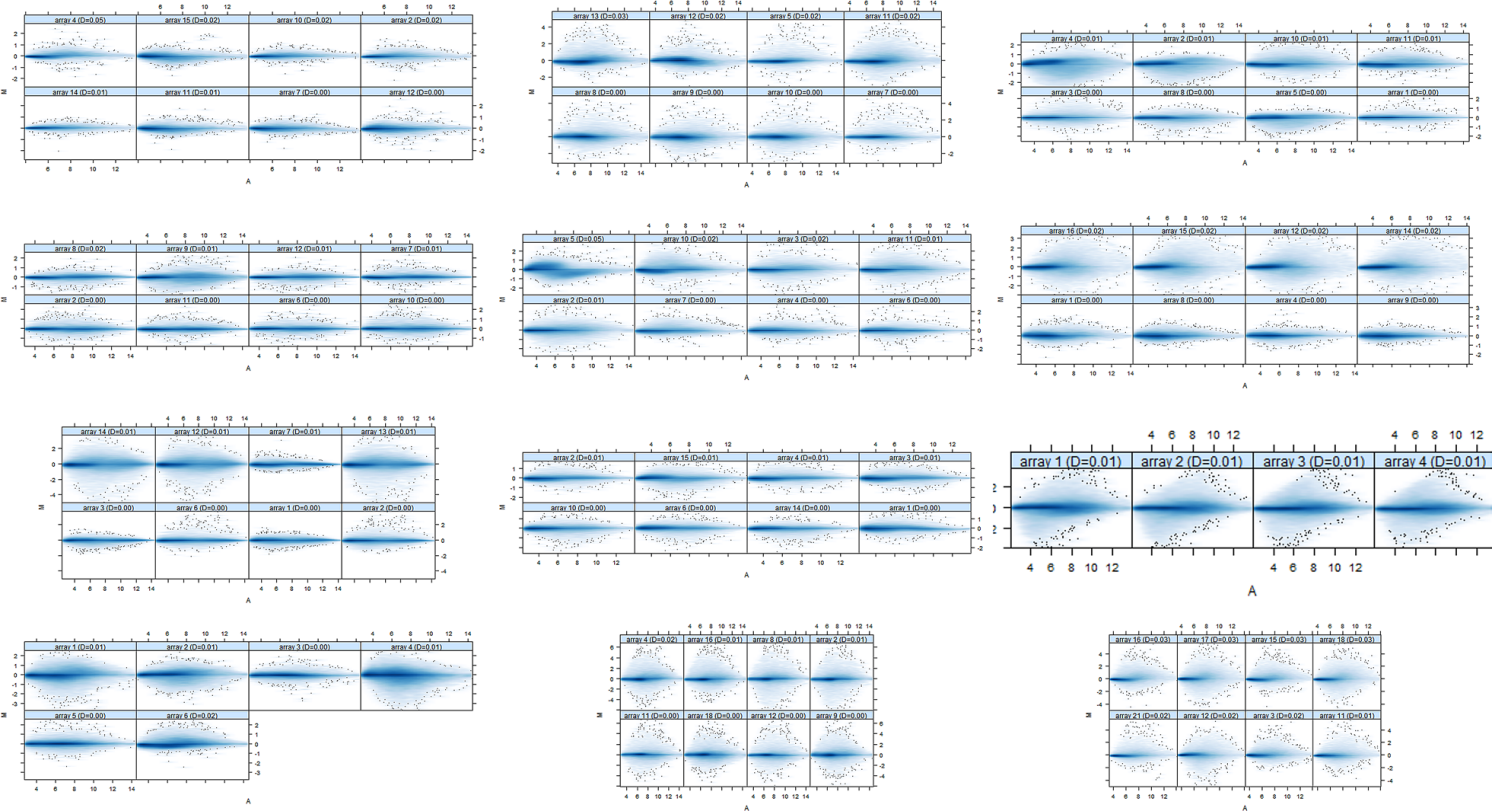
MA plots showing normalization and analysis of quality array metrics. Plots of log intensity ratio (M) vs. log intensity averages (A). Normally, the mass of distribution in the MA plot is expected to be concentrated along the M = 0 axis

**Fig. 2:**
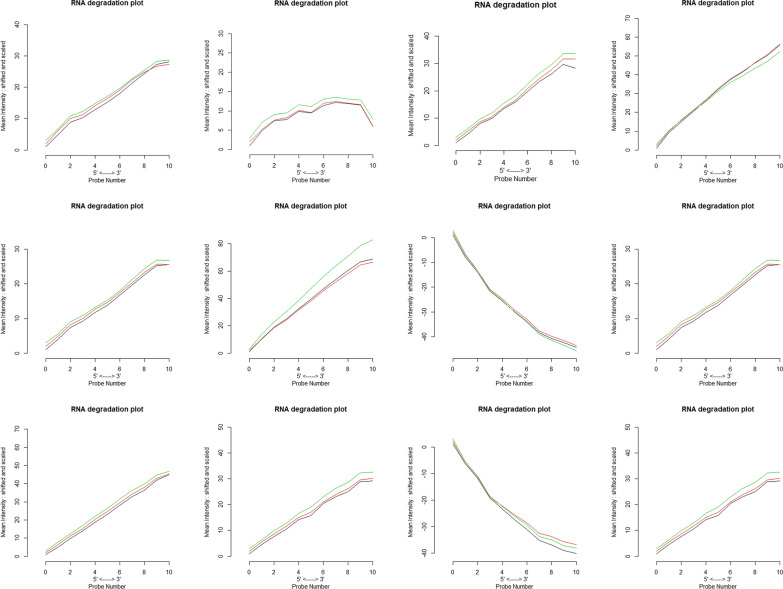
RNA degradation plots produced by plotAffyRNAdeg representing the quality of RNA and its severity of degradation

Twelve breast cancer datasets were downloaded from the GEO database with cell format. Each database was having size of ArrayBatch object 1164 × 1164 and 732 × 732 features with related Affyids (Table 1). Quantile normalization was performed for normalization and background correction. This was done to avoid systematic variation. The probe level data obtained after normalization show the quality of the individual array of each dataset in the MA plots (Fig. 1). The severity of RNA-degradation and significance level was presented by the function plotAffyRNAdeg (Fig. 2) and a single summary statistic for each array in the batch was produced by the function summary of AffyRNAdeg (Additional file 1: Table S1). Additional file 2: Table S2 provides the list of databases, tools, and software used in this study.

**Table 1:** List of cDNA datasets

Dataset Accession No.	Total samples	Tissues	Species	Conditions/type	Platform	Size of arrays	AffyIDs	References
GSE83325	4	Breast cancer	*Homo sapiens*	Control vs. treated	GPL15207 [PrimeView] Affymetrix Human Gene Expression Array	732 × 732 features	49495	[35]
GSE28645	14	Breast cancer	*Homo sapiens*	Control vs. treated	GPL570 [HG-U133_Plus_2] Affymetrix Human Genome U133 Plus 2.0 Array	1164 × 1164 features	54675	[36]
GSE28448	11	Breast cancer	*Homo sapiens*	Control vs. treated	GPL570 [HG-U133_Plus_2] Affymetrix Human Genome U133 Plus 2.0 Array	1164 × 1164 features	54675	[37]
GSE27444	14	Breast cancer	*Homo sapiens*	Control vs. treated	GPL570 [HG-U133_Plus_2] Affymetrix Human Genome U133 Plus 2.0 Array	1164 × 1164 features	54675	[38]
GSE12791	16	Breast cancer	*Homo sapiens*	Control vs. treated	GPL570 [HG-U133_Plus_2] Affymetrix Human Genome U133 Plus 2.0 Array	712 × 712 features	22283	[39]
GSE33658	22	Breast cancer	*Homo sapiens*	Control vs. treated	GPL570 [HG-U133_Plus_2] Affymetrix Human Genome U133 Plus 2.0 Array	1164 × 1164 features	54675	[40]
GSE116781	6	Breast cancer	*Homo sapiens*	Control vs. treated	GPL15207 [PrimeView] Affymetrix Human Gene Expression Array	732 × 732 features	49495	[41]
GSE146911	11	Breast cancer	*Homo sapiens*	Control vs. treated	GPL570 [HG-U133_Plus_2] Affymetrix Human Genome U133 Plus 2.0 Array	1164 × 1164 features	54675	[42]
GSE151635	12	Breast cancer	*Homo sapiens*	Control vs. treated	GPL571 [HG-U133A_2] Affymetrix Human Genome U133A 2.0 Array	732 × 732 features	22277	[43]
GSE71363	18	Breast cancer	*Homo sapiens*	Control vs. treated	GPL570 [HG-U133_Plus_2] Affymetrix Human Genome U133 Plus 2.0 Array	1164 × 1164 features	54675	[44]
GSE99860	16	Breast cancer	*Homo sapiens*	Control vs. treated	GPL570 [HG-U133_Plus_2] Affymetrix Human Genome U133 Plus 2.0 Array	1164 × 1164 features	54675	[45]
GSE99861	16	Breast cancer	*Homo sapiens*	Control vs. treated	GPL570 [HG-U133_Plus_2] Affymetrix Human Genome U133 Plus 2.0 Array	1164 × 1164 features	54675	[46]

### Identification and screening of differentially expressed genes

In each dataset the differential expression analysis provided 50 DEGs by pairwise comparison between biologically comparable groups. Out of these 50 DEGs, the top 24 genes were ranked and selected in each dataset. The selection was based on FDR (< 0.05), *p*-value (≤ 0.05) and |logFC| (> 1) parameters. These 24 DEGs were further shortlisted to eight common genes as potential biomarkers for breast cancer (Additional file 3: Table S3).

### Data curation and cluster analysis

**Fig. 3:**
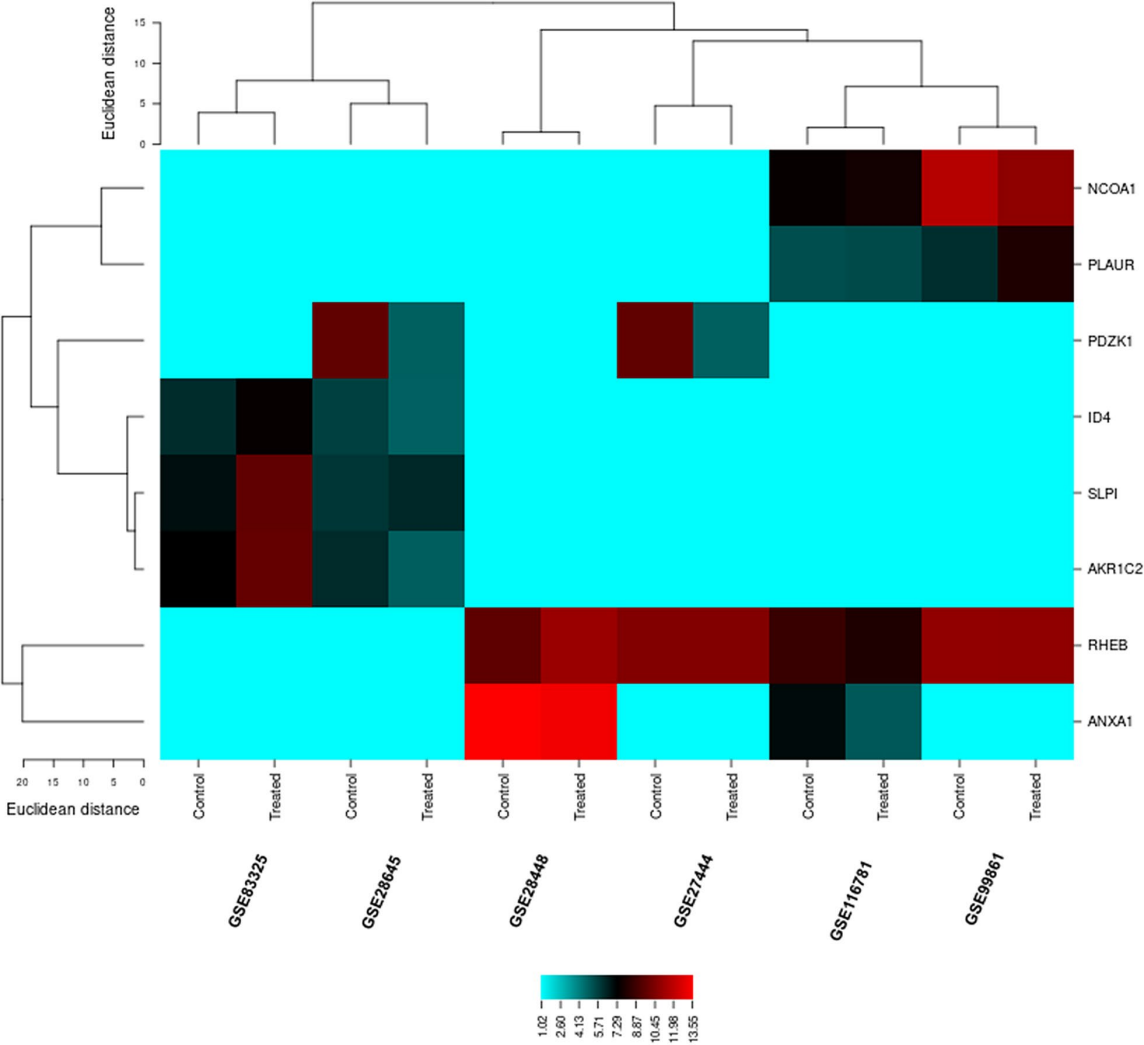
Cluster analysis of breast cancer related differentially expressed genes. Blue represents small distance and red shows large distance. Lines indicate the cluster boundaries in the level of the tree

The gene mapping of 24 DEGs through PubMed, OMIM, MeSH, and PMC databases provided eight significant breast cancer associated genes: ID4, NCOA1, RHEB, PDZK1, PLAUR, AKC1R2, ANXA1 and SLIPI. The role of these genes in breast cancer was curated and counted (Table 2). The genetic expression of breast cancer cell samples showed a clear difference between the control and treated replicates (Fig. 3).

**Table 2:** The differentially expressed breast cancer associated genes curated from Pubmed

Sr. No.	Probe ID	Gene ID	Uniprot_id	Pubmed count	Protein name	Reference link
1	11721688_at	ID4	ID4_HUMAN	50	Inhibitor of DNA binding 4, HLH protein (ID4)	https://pubmed.ncbi.nlm.nih.gov/?term=ID4+and+breast+cancer
2	209106_at	NCOA1	NCOA1_HUMAN	106	Nuclear receptor coactivator 1 (NCOA1)	https://pubmed.ncbi.nlm.nih.gov/?term=ncoa1+and+breast+cancer
3	211924_s_at	PLAUR	UPAR_HUMAN	189	Plasminogen activator, urokinase receptor (PLAUR)	https://pubmed.ncbi.nlm.nih.gov/?term=plaur+and+breast+cancer
4	1555780_a_at	RHEB	RHEB_HUMAN	14	Ras homolog enriched in brain (RHEB)	https://pubmed.ncbi.nlm.nih.gov/?term=rheb+and+breast+cancer
5	205380_at	PDZK1	PDZ1I_HUMAN	26	PDZ domain containing 1 (PDZK1)	https://pubmed.ncbi.nlm.nih.gov/?term=pdzk1+and+breast+cancer
6	11716033_at	SLPI	SLPI_HUMAN	14	Secretory leukocyte peptidase inhibitor (SLPI)	https://pubmed.ncbi.nlm.nih.gov/?term=slpi+and+breast+cancer
7	11729101_a_at	AKR1C2	Q1KXY7_HUMAN	36	Aldo–keto reductase family 1 member C2 (AKR1C2)	https://pubmed.ncbi.nlm.nih.gov/?term=akr1c2+and+breast+cancer
8	201012_at	ANXA1	ANXA1_HUMAN	52	Annexin A1 (ANXA1)	https://pubmed.ncbi.nlm.nih.gov/?term=anxa1+and+breast+cancer

### miRNA target prediction analysis

The computational algorithms (miRDB) identified multiple breast cancer associated miRNA targets for each gene such as hsa-miR-650, hsa-miR-203a-3p, hsa-miR-4520-3p, hsa-miR-1185-1-3p, hsa-miR-15b-3p and hsa-miR-942-5p. The dysregulation of these signature genes is linked to the progression of breast cancer. The genes ID4, RHEB, AKR1C2, ANXA1 and PDZK1 predicted 191, 74, 108, 41 and 41 miRNAs hits, respectively (Table 3).

**Table 3:** miRNA targets of breast cancer related genes

Uniprot id	Gene symbol	miRNA	Target score	Total miRNA hits	Structure of predicted duplex
NCOA1_HUMAN	NCOA1	hsa-miR-650	99	205	AGGAGGCAGCGCUCUCAGGAC
ID4_HUMAN	ID4	hsa-miR-203a-3p	98	191	GUGAAAUGUUUAGGACCACUAG
RHEB_HUMAN	RHEB	hsa-miR-4520-3p	96	74	UUGGACAGAAAACACGCAGGAA
ANXA1_HUMAN	ANXA1	hsa-miR-1185-1-3p	93	41	AUAUACAGGGGGAGACUCUUAU
PDZ1I_HUMAN	PDZK1	hsa-miR-15b-3p	92	41	CGAAUCAUUAUUUGCUGCUCUA
UPAR_HUMAN	PLAUR	hsa-miR-942-5p	92	43	UCUUCUCUGUUUUGGCCAUGUG
Q1KXY7_HUMAN	AKR1C2	hsa-miR-185-5p	90	108	UGGAGAGAAAGGCAGUUCCUGA
SLPI_HUMAN	SLPI	hsa-miR-3173-3p	72	8	AAAGGAGGAAAUAGGCAGGCCA

### Protein network analysis

**Fig. 4:**
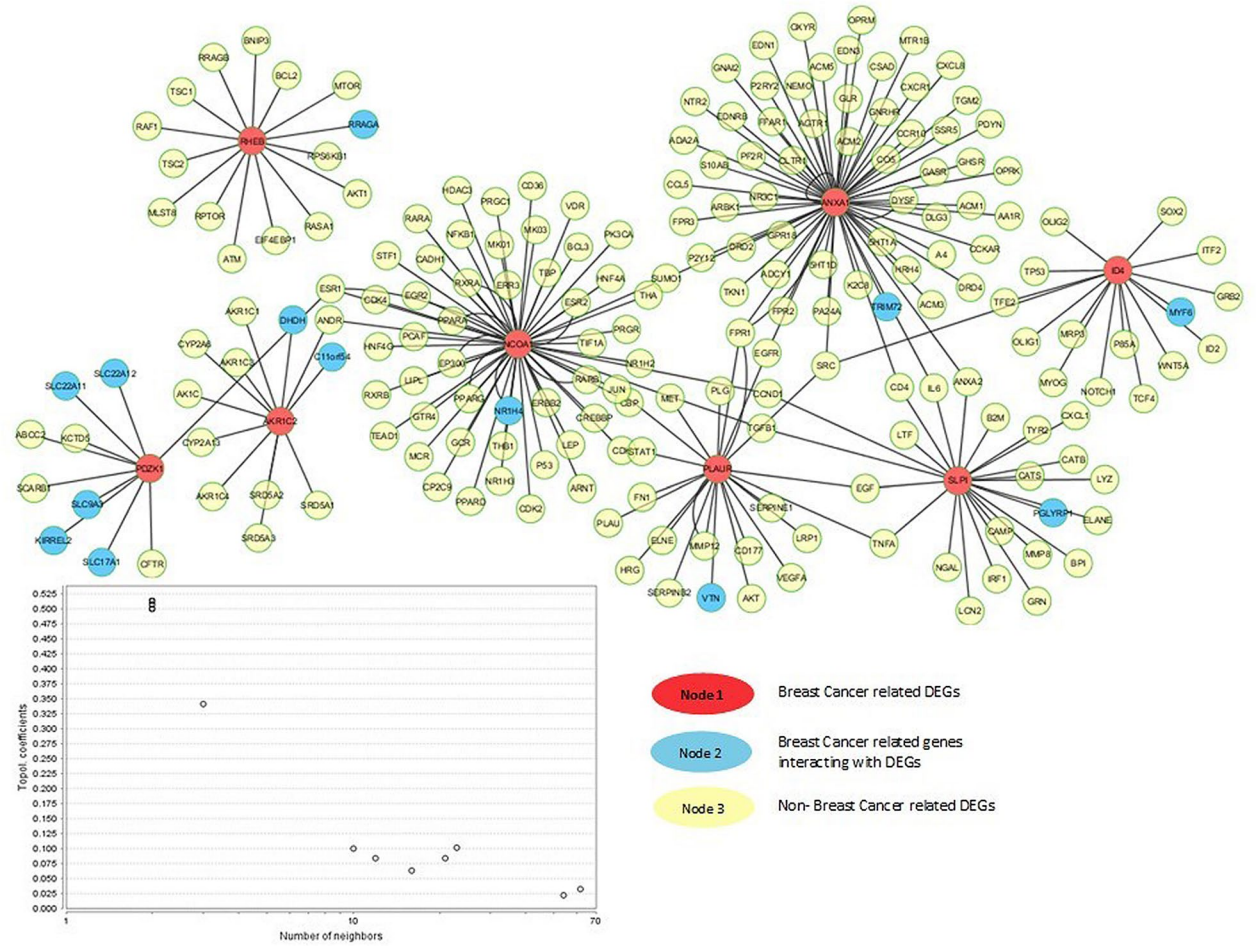
Gene network of breast cancer related differentially expressed genes with 207 nodes and 226 edges. Red and blue nodes indicate the breast cancer associated potential biomarkers while yellow nodes represent the non-breast cancer target proteins

**Fig. 5:**
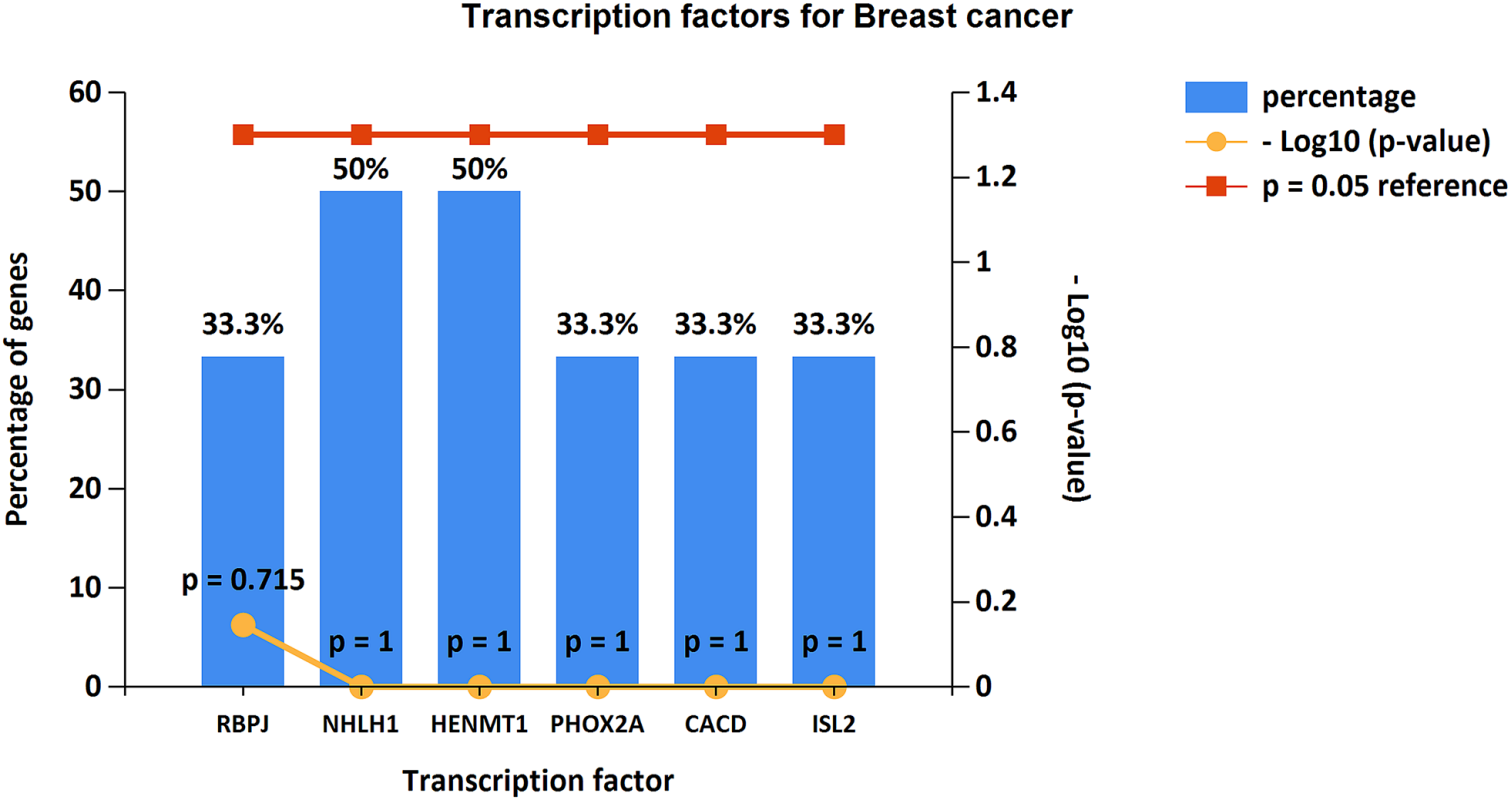
Transcription factors for breast cancer associated gene signatures involved to alter gene expression in a host cell to promote breast cancer resistance and progression

The protein–protein interaction analysis revealed the interaction of breast cancer related genes with other potential genes contributing to a pathological phenotype. The network showed a total of 207 nodes and 226 edges that were retrieved from STRING [14] and HAPPI [15] databases. The network was categorized in three neighborhoods: red and blue nodes indicate the breast cancer associated potential biomarkers while the remaining yellow nodes represent the non-breast cancer target proteins. The potential biomarkers were found to functionally interact with other biologically essential target proteins, some of which are TCF4, TP53, mTOR, NOTCH1, ESR1 and ESR2. The source protein ID4 showed interaction with TCF4, NOTCH1 and WNT while NCOA1 and PDZK1 interacted with ESR1 and ESR2 potential biomarker proteins. ANXA1 was also associated with the CCL5, CXCR 10 and CXCL8 family of cytokines. The network analyzer was used to analyze the topological properties of the network. It also helped in classifying and improving the network performance (Fig. 4). The disease gene mapping of target genes using CTD showed that more than 50 genes have a functional relation with the source/seeder genes in breast cancer (Fig. 5). In gene enrichment analysis, the targeted genes were selected based on fold change and a *p*-value cut-off (< 0.05). The analysis revealed significant enrichment of these genes with mTOR signaling pathway, TGF-β signaling pathway, P13-AKT signaling pathway, insulin signaling pathway, thyroid signaling pathway and complement coagulation cascade (Table 4). The transcription factors identified were RBPJ, NHLH1, HENMT1, PHOX2A, CACD and ISL2. The transcription factors (TFs) NHLH1 and HENMT1 showed 50% abundance with known breast cancer genes (Fig. 5).

**Table 4:** Pathway enrichment and gene ontology of breast cancer related DEGs

Category	Term	Count	*p*-value
GOTERM_BP_FAT	Positive regulation of cell differentiation	4	3.9 × 10^−3^
GOTERM_BP_FAT	Gliogenesis	3	4.1 × 10^−3^
GOTERM_BP_FAT	Rhythmic process	3	6.9 × 10^−3^
GOTERM_BP_FAT	Cellular lipid metabolic process	4	7.1 × 10^−3^
GOTERM_BP_FAT	Estrous cycle	2	7.1 × 10^−3^
GOTERM_BP_FAT	Epithelium development	4	7.4 × 10^−3^
GOTERM_BP_FAT	Negative regulation of hydrolase activity	3	1.1 × 10^−2^
GOTERM_BP_FAT	Response to drug	3	1.2 × 10^−2^
GOTERM_BP_FAT	Regulation of oligodendrocyte differentiation	2	1.2 × 10^−2^
GOTERM_BP_FAT	Reproductive structure development	3	1.2 × 10^−2^
GOTERM_BP_FAT	Gland development	3	1.2 × 10^−2^
GOTERM_BP_FAT	Prostaglandin metabolic process	2	1.3 × 10^−2^
GOTERM_BP_FAT	Lipid metabolic process	4	1.4 × 10^−2^
GOTERM_BP_FAT	Neurogenesis	4	1.8 × 10^−2^
GOTERM_BP_FAT	Prostate gland development	2	1.9 × 10^−2^
GOTERM_BP_FAT	Positive regulation of cell death	3	2.5 × 10^−2^
GOTERM_CC_FAT	Extracellular exosome	5	5.9 × 10^−3^
GOTERM_CC_FAT	Extracellular vesicle	5	6.0 × 10^−3^
GOTERM_CC_FAT	Membrane-bound vesicle	5	1.5 × 10^−2^
GOTERM_CC_FAT	Extracellular region	5	3.8 × 10^−2^
GOTERM_CC_FAT	Extrinsic component of membrane	2	8.8 × 10^−2^
GOTERM_MF_FAT	Receptor binding	4	2.2 × 10^−2^
GOTERM_MF_FAT	Enzyme binding	4	3.8 × 10^−2^
GOTERM_MF_FAT	Protein dimerization activity	3	9.2 × 10^−2^

### Pathway modeling

**Fig. 6:**
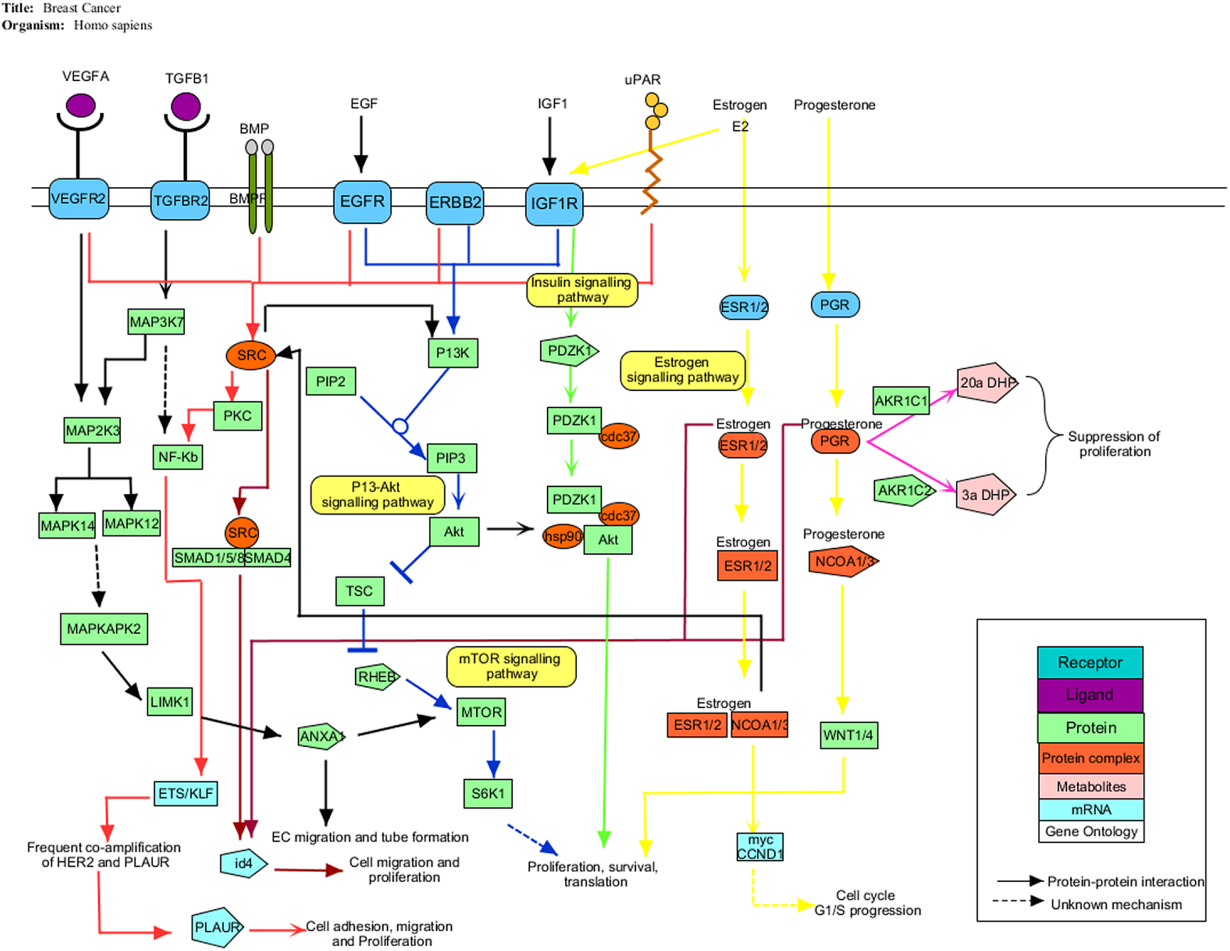
Pathway analysis. Integrated gene signaling pathways involved in the progression of breast cancer. Gene signatures were mapped on KEGG pathways for signaling and metabolic reconstruction

The gene signatures isolated were further studied to understand their role in the progression of breast cancer and their underlying molecular mechanism. The signature genes were analyzed for their interaction with other proteins in breast carcinogenesis through reconstruction of a network. The pathways involved in the progression of breast cancer were the MTOR signaling pathway, estrogen signaling pathway, P13-AKT signaling pathway, TGF-β signaling pathway and the insulin signaling pathway. The source genes interact with other target genes through these signaling pathways leading to the occurrence of breast cancer. The network shows the heterogeneous nature of breast cancer which is the major obstacle in defining therapies with desirable outcomes (Fig. 6).

### Drug-gene network analysis

**Fig. 7:**
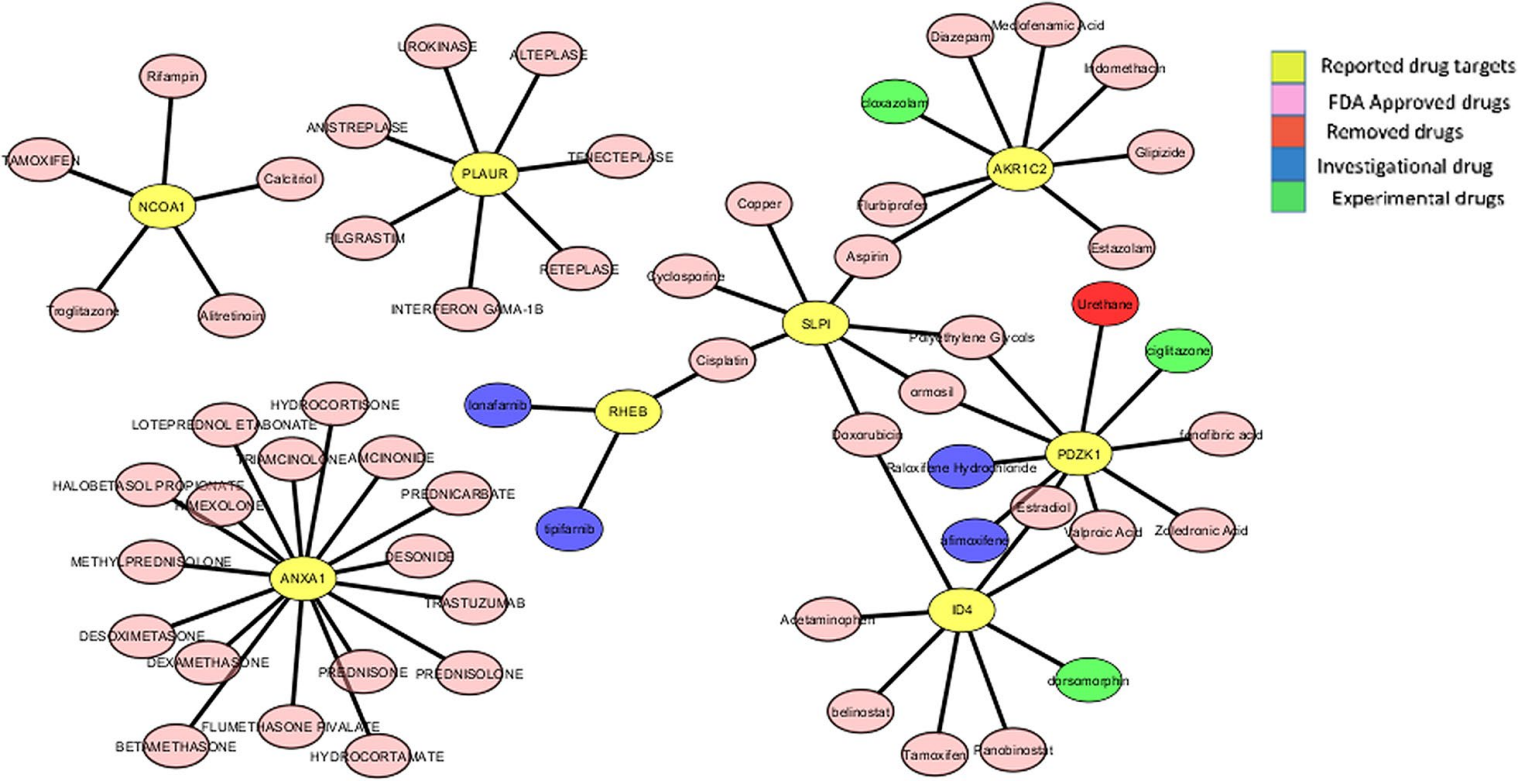
Drug-gene network. Drug-gene network constructed between the reported drugs and their target signature genes showing 66 nodes and 65 edges. Color codes are given in the legends. The drug-gene network shows potential drug targets for signature genes by curating using PMC, CTD and Drug Bank databases

For drug-gene network analysis the toxicogenomic approach was used to further investigate the existing treatment options for breast cancer therapy. This was done to better understand the disease etiology. The publicly available database CTD identified 65 drugs that interacted with these signature genes. In total, 57 target drugs were FDA approved (Table 5). These drugs were found to interact with signature genes that are involved in the progression of breast cancer (Fig. 7).

**Table 5:** Drug targets of identified differentially expressed genes

Gene	Drugs	Drug Bank ID	FDA approval
PDZK1	Afimoxifene	DB04468	Investigational
PDZK1	Raloxifene hydrochloride	DB00481	Investigational
PDZK1	Urethane	DB04827	Removed
PDZK1	Valproic acid	DB00313	Approved
PDZK1	Ciglitazone	DB09201	Experimental
PDZK1	Estradiol	DB00783	Approved
PDZK1	Fenofibric acid	DB13873	Approved
PDZK1	Ormosil	DB00742	Approved
PDZK1	Polyethylene glycols	DB09287	Approved
PDZK1	Zoledronic acid	DB00399	Approved
ID4	Acetaminophen	DB00316	Approved
ID4	Belinostat	DB05015	Approved
ID4	Dorsomorphin	DB08597	Experimental
ID4	Doxorubicin	DB00997	Approved
ID4	Estradiol	DB00783	Approved
ID4	Panobinostat	DB06603	Approved
ID4	Tamoxifen	DB00675	Approved
ID4	Valproic acid	DB00313	Approved
RHEB	Cisplatin	DB00515	Approved
RHEB	Lonafarnib	DB06448	Investigational
RHEB	Tipifarnib	DB04960	Investigational
SLPI	Copper	DB09130	Approved
SLPI	Ormosil	DB00742	Approved
SLPI	Polyethylene glycols	DB09287	Approved
SLPI	Doxorubicin	DB00997	Approved
SLPI	Cyclosporine	DB00091	Approved
SLPI	Cisplatin	DB00515	Approved
SLPI	Aspirin	DB00945	Approved
AKR1C2	Aspirin	DB00945	Approved
AKR1C2	Cloxazolam	DB01553	Experimental
AKR1C2	Diazepam	DB00829	Approved
AKR1C2	Estazolam	DB01215	Approved
AKR1C2	Flurbiprofen	DB00712	Approved
AKR1C2	Glipizide	DB01067	Approved
AKR1C2	Indomethacin	DB00328	Approved
AKR1C2	Meclofenamic acid	DB00939	Approved
NCOA1	Tamoxifen	DB00675	Approved
NCOA2	Calcitriol	DB00136	Approved
NCOA3	Rifampin	DB01045	Approved
NCOA4	Troglitazone	DB00197	Approved
NCOA5	Alitretinoin	DB00523	Approved
PLAUR	Urokinase	DB00013	Approved
PLAUR	Tenecteplase	DB00031	Approved
PLAUR	Anistreplase	DB00029	Approved
PLAUR	Filgrastim	DB00099	Approved
PLAUR	Interferon gama-1b	DB00011	Approved
PLAUR	Reteplase	DB00015	Approved
PLAUR	Alteplase	DB00009	Approved
ANXA1	Desonide	DB01260	Approved
ANXA1	Prednisone	DB00635	Approved
ANXA1	Trastuzumab	DB00072	Approved
ANXA1	Loteprednol etabonate	DB14596	Approved
ANXA1	Desoximetasone	DB00547	Approved
ANXA1	Hydrocortisone	DB00741	Approved
ANXA1	Hydrocortamate	DB00769	Approved
ANXA1	Triamcinolone	DB00620	Approved
ANXA1	Prednisolone	DB00860	Approved
ANXA1	Amcinonide	DB00288	Approved
ANXA1	Flumethasone pivalate	DB00663	Approved
ANXA1	Betamethasone	DB00443	Approved
ANXA1	Methylprednisolone	DB00959	Approved
ANXA1	Rimexolone	DB00896	Approved
ANXA1	Halobetasol propionate	DB00596	Approved
ANXA1	Dexamethasone	DB01234	Approved
ANXA1	Prednicarbate	DB01130	Approved

## Discussion

Due to its recurrence and heterogeneous nature, breast cancer is the leading cause of death in women globally. This calls for a better understanding of the molecular mechanisms of breast cancer in order to improve diagnosis and management.

This study focuses on the identification of several gene signatures, their functional annotation, potential protein–protein interactions and reconstruction of biological pathways for a better understanding of the disease. The differential expression analysis revealed eight gene signatures out of 50 DEGs based on physicochemical and functional studies that play a role in breast carcinogenesis. ID4, NOCA1, RHEB, ANXA1, AKR1C2, PDZK1, PLAUR and SLPI are the identified DEGs out of which five are upregulated and three are downregulated. The gene ontology of these genes showed functional enrichment in cellular communication, signal transduction, protein metabolism, transport and steroid hormone receptor signaling as well as essential roles in several important signaling pathways such as the MTOR, TGF-B, P13-Akt and insulin. These pathways have been studied for their role in the progression and occurrence of several cancers. ID4 belongs to a family of four helix-loop-helix (HLH) transcriptional regulators, termed as inhibitors of differentiation (ID) proteins. These proteins are involved in the regulation of several cell processes such as differentiation, transcription and cell cycle progression. Emerging evidence has shown a proto-oncogenic role of ID4 in basal like breast cancer (BLBC). An overexpression of this gene is observed in this subtype of breast cancer and is correlated with the expression of TP53 protein which is involved in higher grade and metastasis risk. This has led it to be a poor prognostic marker of BLBC as the proliferation of BLBC cell lines require an overexpression of ID4 [26]. The gene network analysis also revealed the interaction of ID4 with several other proteins such as TCF, WNT, TP53 and NOTCH1. This supports the previous evidence of correlation of ID4 with TP53 in the proliferation of BLBC.

The overexpression of nuclear receptor coactivator 1 (NCOA1) has also shown a positive correlation with disease metastasis and recurrence that resides in a subset of breast cancers. This gene belongs to the p160 SRC family and interacts with certain nuclear receptors and transcription factors (TFs) playing important roles in growth, development, reproduction and metabolism as well as in cancer. NCOA1 has been associated with HER2 expression, metastasis, disease recurrence and poor survival and overexpression in 19–29% of breast tumors [27]. Other interacting proteins identified through network analysis showing crosstalk with NCOA1 are the ESR and PPAR (Fig. 5). The pathway analysis also clarifies the role of NCOA1 in proliferation and metastasis of breast cancer by interaction with these proteins (Fig. 7). Another source protein identified through differential expression analysis is PDZ domain containing 1 (PDZK1) which is an adaptor protein expressed in the proximal tubules of kidney and has a pivotal role in lipid metabolism. However, this protein is thought to be responsive to estrogen in breast cancer cell lines (mcf-7). A significant correlation between 17B-estradiol plasma levels and PDZK1 mRNA expression has been shown in ER-α (+) breast tumors providing a link between Er-α and PDZK1 [28]. A potential candidate involved in the indirect link of this association is insulin-like growth factor-1 (IGS-1R). The gene ontology studies of these genes also revealed enrichment of these genes in the insulin signaling pathway, suggesting a link of this pathway in cell proliferation of breast tumors.

Ras human enriched in brain (Rheb) is a small GTP-binding protein and a well-known regulator of mTOR. mTOR plays a pivotal role in cell proliferation, aging, protein synthesis and autophagy. Recent evidence has suggested a hyperactivity in Rheb-mTORC1 signaling axis in several human carcinomas [29]. Evidence also suggest an elevated expression of RHEB in epithelial cells of fibroadenomas providing an association of RHEB with insulin/AKT/TOR signaling pathway in benign tumor development [30]. The pathway analysis has also shown association of Rheb with these proteins suggesting its important role in cell cycle control and cell growth. Secreted proteins play a pivotal role in several types of cancer metastasis including breast tumors. One of the secreted proteins identified through differential analysis is SLPI which has a role in the progression and development of tumors. Several tumors have shown elevated gene expression levels of SLPI such as ovarian and lung cancer. A recent study has identified SLPI as a new target for anti-metastatic therapies due to its pro-metastatic part of secretome for breast cancer, chiefly for TNCs [31]. The two aldo–keto reductases AKR1C1 and AKR1C2 belong to the super family of AKR1C1 and are involved in progesterone metabolism. The metabolites of progesterone are basically involved in suppression of cell proliferation and adhesion. In tumorous breast tissues the expression of AKR1C1 and AKR1C2 is reduced promoting tumor growth and progression [32]. The association of over-activation of PLAUR (uPAR) with increased aggressive carcinoma is also well-studied. A correlation has been observed between HER2 and uPAR mRNA in disseminated tumors suggesting a cross talk between HER2 and uPAR signaling pathways causing recurrence or metastasis [33]. Moreover, Annexin A1 (AnxA1) is also a candidate regulator of oncogenic switch during which cancer cells change their phenotype from epithelial to migratory, mesenchymal-like. AnxA1 is an actin regulatory protein and its overexpression is associated with the BLBC subtype. It has a pro-angiogenic role in vascular endothelial cells, tumor growth and metastasis and is also involved in the regulation of TGFβ signaling. Evidence suggests AnxA1 as an additional marker in discriminating BLBC diagnosis from other subtypes [34]. The drug-gene network analysis revealed that several common drugs have shown interactions with these signature genes such as Tamoxifen, Cisplatin, Diazepam, Aspirin, Hydrocortisone, etc. opening the platform for repurposing of these drugs to better manage this disease.

## Conclusion

This study has opened new insights for potential targets for breast cancer, their relations with other signaling proteins and their involvement in the progression and development of breast cancer through cross talk. The pathway analysis further clarifies the role of several genes and contributes to the efficient management of this disease.

## Supplementary Information

**Additional file 1: Table S1.** The function summaryAffyRNAdeg of Bioconductor package produced a single summary-statistic for each array in the batch dataset.

**Additional file 2: Table S2.** List of Databases, software, and Tools used in this study.

**Additional file 3: Table S3.** Preliminary investigation of common and related differentially expressed genes of each microarray dataset.

## Data Availability

The data has been presented with the article.

## References

[1] Friedenreich CM (2011). Physical activity and breast cancer: review of the epidemiologic evidence and biologic mechanisms. Recent Results Cancer Res Fortschritte der Krebsforschung Progres dans les recherches sur le cancer.

[2] Siegel R, Naishadham D, Jemal A (2013). Cancer statistics, 2013. CA Cancer J Clin.

[3] Lynch HT, Lynch JF (1986). Breast cancer genetics in an oncology clinic: 328 consecutive patients. Cancer Genet Cytogenet.

[4] Sharif S, Moran A, Huson S, Iddenden R, Shenton A, Howard E (2007). Women with neurofibromatosis 1 (nf1) are at a moderately increased risk of developing breast cancer and should be considered for early screening. J Med Genet.

[5] Guarino M, Rubino B, Ballabio G (2007). The role of epithelial–mesenchymal transition in cancer pathology. Pathology.

[6] Ilyas U, uz Zaman S, Altaf R, Nadeem H, Muhammad SA (2020). Genome wide meta-analysis of cDNA datasets reveals new target gene signatures of colorectal cancer based on systems biology approach. J Biol Res Thessaloniki.

[7] Obenchain V, Lawrence M, Carey V, Gogarten S, Shannon P, Morgan M (2014). VariantAnnotation: a bioconductor package for exploration and annotation of genetic variants. Bioinformatics.

[8] Troyanskaya O, Cantor M, Sherlock G, Brown P, Hastie T, Tibshirani R (2001). Missing value estimation methods for DNA microarrays. Bioinformatics.

[9] Fasold M, Binder H (2013). AffyRNADegradation: control and correction of RNA quality effects in GeneChip expression data. Bioinformatics.

[10] Ferreira J, Zwinderman A (2006). On the Benjamini–Hochberg method. Ann Stat.

[11] Jin Y, Da W (2013). RETRACTED ARTICLE: screening of key genes in gastric cancer with DNA microarray analysis. Eur J Med Res.

[12] Eisen MB, Spellman PT, Brown PO, Botstein D (1998). Cluster analysis and display of genome-wide expression patterns. Proc Natl Acad Sci.

[13] Scherf U, Ross DT, Waltham M, Smith LH, Lee JK, Tanabe L (2000). A gene expression database for the molecular pharmacology of cancer. Nat Genet.

[14] Szklarczyk D, Franceschini A, Kuhn M, Simonovic M, Roth A, Minguez P (2011). The STRING database in 2011: functional interaction networks of proteins, globally integrated and scored. Nucleic Acids Res.

[15] Chen JY, Mamidipalli S, Huan T (2009). HAPPI: an online database of comprehensive human annotated and predicted protein interactions. BMC Genom.

[16] Shannon P, Markiel A, Ozier O, Baliga NS, Wang JT, Ramage D (2003). Cytoscape: a software environment for integrated models of biomolecular interaction networks. Genome Res.

[17] Huang DW, Sherman BT, Tan Q, Kir J, Liu D, Bryant D (2007). DAVID bioinformatics resources: expanded annotation database and novel algorithms to better extract biology from large gene lists. Nucleic Acids Res.

[18] Pathan M, Keerthikumar S, Ang CS, Gangoda L, Quek CY, Williamson NA (2015). FunRich: an open access standalone functional enrichment and interaction network analysis tool. Proteomics.

[19] Nam D, Kim S-Y (2008). Gene-set approach for expression pattern analysis. Brief Bioinform.

[20] Muhammad SA, Ahmed S, Ali A, Huang H, Wu X, Yang XF (2014). Prioritizing drug targets in *Clostridium botulinum* with a computational systems biology approach. Genomics.

[21] Alshalalfa M, Alhajj R (2013). Using context-specific effect of miRNAs to identify functional associations between miRNAs and gene signatures. BMC Bioinform.

[22] Kanehisa M, Goto S (2000). KEGG: kyoto encyclopedia of genes and genomes. Nucleic Acids Res.

[23] Kutmon M, van Iersel MP, Bohler A, Kelder T, Nunes N, Pico AR (2015). PathVisio 3: an extendable pathway analysis toolbox. PLoS Comput Biol.

[24] Davis AP, Rosenstein MC, Wiegers TC, Mattingly CJ (2011). DiseaseComps: a metric that discovers similar diseases based upon common toxicogenomic profiles at CTD. Bioinformation.

[25] Wishart DS, Knox C, Guo AC, Shrivastava S, Hassanali M, Stothard P (2006). DrugBank: a comprehensive resource for in silico drug discovery and exploration. Nucleic Acids Res.

[26] Baker LA, Holliday H, Swarbrick A (2016). ID4 controls luminal lineage commitment in normal mammary epithelium and inhibits BRCA1 function in basal-like breast cancer. Endocr Relat Cancer.

[27] Qin L, Wu Y-L, Toneff MJ, Li D, Liao L, Gao X (2014). NCOA1 directly targets M-CSF1 expression to promote breast cancer metastasis. Can Res.

[28] Kim H, Abd Elmageed ZY, Davis C, El-Bahrawy AH, Naura AS, Ekaidi I (2014). Correlation between PDZK1, Cdc37, Akt and breast cancer malignancy: the role of PDZK1 in cell growth through Akt stabilization by increasing and interacting with Cdc37. Mol Med.

[29] He L, Ren Y, Zheng Q, Wang L, Lai Y, Guan S (2016). Fas-associated protein with death domain (FADD) regulates autophagy through promoting the expression of Ras homolog enriched in brain (Rheb) in human breast adenocarcinoma cells. Oncotarget.

[30] Eom M, Han A, Lee MJ, Park KH (2012). Expressional difference of RHEB, HDAC1, and WEE1 proteins in the stromal tumors of the breast and their significance in tumorigenesis. Korean J Pathol.

[31] Kozin SV, Maimon N, Wang R, Gupta N, Munn L, Jain RK (2017). Secretory leukocyte protease inhibitor (SLPI) as a potential target for inhibiting metastasis of triple-negative breast cancers. Oncotarget.

[32] Wenners A, Hartmann F, Jochens A, Roemer AM, Alkatout I, Klapper W (2016). Stromal markers AKR1C1 and AKR1C2 are prognostic factors in primary human breast cancer. Int J Clin Oncol.

[33] Chandran VI, Eppenberger-Castori S, Venkatesh T, Vine KL, Ranson M (2015). HER2 and uPAR cooperativity contribute to metastatic phenotype of HER2-positive breast cancer. Oncoscience.

[34] de Graauw M, van Miltenburg MH, Schmidt MK, Pont C, Lalai R, Kartopawiro J (2010). Annexin A1 regulates TGF-beta signaling and promotes metastasis formation of basal-like breast cancer cells. Proc Natl Acad Sci USA.

[35] Kong S-Y, Kim K-S, Kim J, Kim MK, Lee KH, Lee J-Y (2016). The ELK3-GATA3 axis orchestrates invasion and metastasis of breast cancer cells in vitro and in vivo. Oncotarget.

[36] McCartan D, Bolger JC, Fagan A, Byrne C, Hao Y, Qin L (2012). Global characterization of the SRC-1 transcriptome identifies ADAM22 as an ER-independent mediator of endocrine-resistant breast cancer. Can Res.

[37] Hesling C, Fattet L, Teyre G, Jury D, Gonzalo P, Lopez J (2011). Antagonistic regulation of EMT by TIF1γ and Smad4 in mammary epithelial cells. EMBO Rep.

[38] Lee J, Hirsh AS, Wittner BS, Maeder ML, Singavarapu R, Lang M (2011). Induction of stable drug resistance in human breast cancer cells using a combinatorial zinc finger transcription factor library. PLoS ONE.

[39] Luo W, Schork NJ, Marschke KB, Ng S-C, Hermann TW, Zhang J (2011). Identification of polymorphisms associated with hypertriglyceridemia and prolonged survival induced by bexarotene in treating non-small cell lung cancer. Anticancer Res.

[40] Massarweh S, Tham YL, Huang J, Sexton K, Weiss H, Tsimelzon A (2011). A phase II neoadjuvant trial of anastrozole, fulvestrant, and gefitinib in patients with newly diagnosed estrogen receptor positive breast cancer. Breast Cancer Res Treat.

[41] Cui B, Luo Y, Tian P, Peng F, Lu J, Yang Y (2019). Stress-induced epinephrine enhances lactate dehydrogenase A and promotes breast cancer stem-like cells. J Clin Invest.

[42] Jayaraman S, Hou X, Kuffel MJ, Suman VJ, Hoskin TL, Reinicke KE (2020). Antitumor activity of Z-endoxifen in aromatase inhibitor-sensitive and aromatase inhibitor-resistant estrogen receptor-positive breast cancer. Breast Cancer Res.

[43] Hakim S, Craig JM, Koblinski JE, Clevenger CV (2020). Inhibition of the activity of cyclophilin A impedes prolactin receptor-mediated signaling, mammary tumorigenesis, and metastases. Iscience.

[44] Sayar N, Karahan G, Konu O, Bozkurt B, Bozdogan O, Yulug IG (2015). Transgelin gene is frequently downregulated by promoter DNA hypermethylation in breast cancer. Clin Epigenet.

[45] Marchan R, Büttner B, Lambert J, Edlund K, Glaeser I, Blaszkewicz M (2017). Glycerol-3-phosphate acyltransferase 1 promotes tumor cell migration and poor survival in ovarian carcinoma. Can Res.

[46] Lesjak MS, Marchan R, Stewart JD, Rempel E, Rahnenführer J, Hengstler JG (2014). EDI3 links choline metabolism to integrin expression, cell adhesion and spreading. Cell Adhes Migr.

